# Territory holders and non-territory holders in Ayu fish coexist only in the population growth process due to hysteresis

**DOI:** 10.1038/s41598-017-16859-4

**Published:** 2017-12-01

**Authors:** Yuki Katsumata, Takashi Uehara, Hiromu Ito, Jin Yoshimura, Kei-ichi Tainaka, Genki Ichinose

**Affiliations:** 1 Department of Mathematical and Systems Engineering, Shizuoka University, Hamamatsu, Shizuoka, 432-8561 Japan; 2 Department of Computer Science, School of Computing, Tokyo Institute of Technology, Yokohama, Kanagawa 226-8502 Japan; 3 Department of Preschool Education, Nagoya College, 48 Takeji, Sakae-cho, Toyoake, Aichi 470-1193 Japan; 4 Department of International Health, Institute of Tropical Medicine, Nagasaki University, Nagasaki, 852-8523 Japan; 5 Graduate School of Science and Technology, Shizuoka University, Hamamatsu, Shizuoka, 432-8561 Japan; 6 Department of General Systems Studies, University of Tokyo, Meguro, Tokyo, 153-8902 Japan; 7 Department of Environmental and Forest Biology, State University of New York College of Environmental Science and Forestry, Syracuse, NY, 13210 USA; 8 Marine Biosystems Research Center, Chiba University, Uchiura, Kamogawa, Chiba, 299-5502 Japan

## Abstract

Ayu fish form feeding territories during a non-breeding (growing) season. When the density of the fish increases, phases gradually change. In the early growing season, all fish can hold territories at low density. Once all territory sites are occupied, newcomers become floaters. As the density further increases, territory holders have to spend much more time in defending their own territory and lose the time to feed on algae. Eventually, all fish give up their own territories and then form a school. In contrast, when the density decreases, territories are directly reformed from the school. In short, ayu fish exhibit a different transition, called hysteresis, where the two transitions occur widely-apart from each other. The dynamics of this intrinsic phenomena has not been demonstrated in previous studies. We develop a rate equation to describe the population dynamics within territorial competition. Our model successfully reproduces territorial hysteresis and indicates that territory holders and floaters can coexist only in the process of population growth. Moreover, we also find that the two critical densities of territorial hysteresis are conspicuously different from each other when the increase of the density of floaters sharply influences (step-function-like) the territories.

## Introduction

Territoriality and group foraging are classical examples of behavioral strategies to adapt to different ecological circumstances^1^. These strategies are two different ways of adaptation^2^. Territorial behavior is an adaptation of solitary animals^3–5^ while group foraging is an adaptation of animals living as a group^6,7^. These two different evolutionary adaptations may occur in closely related species^2^. For example, in most migratory birds, mating pairs often form breeding territories while they forage as groups during non-breeding (growing) seasons. However, it is extremely rare to see a transition between territoriality and group foraging in a single season. Here, we provide a unique case study of fish which exhibit both territoriality and group foraging (school) as adaptive responses during a single season.

Ayu fish (*Plecoglossus altivelis*, Osmeridae) are an endemic migratory fish in Japan^8–10^. This fish has a distinctive life as follows^8,9,11^. The life cycle of ayu is completed in one year. In autumn (late August–early September), eggs are spawned downstream of river hatch. The hatched larvae drift to an estuary near the sea within a few days and mostly feed on zooplankton and small aquatic insects. In spring (April–May), juvenile fish migrate to midstream (and/or upstream) of a river, where algae (diatoms) grow on rocks and stones of riverbeds in rapids (swift current). They feed on these algae from spring to fall. In this stage, especially large fish can hold their own territories in rapids, and territorial competition for food violently occurs between territory holders and non-territory holders (floaters). In fall, when ayu fish mature, they swim downstream together. They spawn eggs and die soon afterwards downstream of a river. Thus, ayu is a diadromous fish^11^.

Ayu exhibit a historical effect (hysteresis): the breakdown and formation processes of territory are greatly different. When densities of fish increase, phases gradually change as follows. In early growing season (May), all fish can hold their own algae-feeding territories at low density^12^. This feeding territory is formed in rapids where diatoms grow on the surface of rocks^13,14^. During this growing season, many fish swim together and fish density increases day by day. Once all the rapids are occupied, surplus fish cannot form territories. These fish become floaters and stay in (deep) pools. In contrast to rapids, pools without algae are not suitable for ayu because floaters can only feed on a small amount of algae and insects which drift from the rapid. Because floaters cannot sufficiently feed in pools, they often intrude into other’s territories to steal algae. Territory holders violently attack floaters in order to defend their own territories. As the density of fish further increases and exceeds the critical limit, all fish give up their own territories and form a school. In contrast, when the density decreases and falls below the other critical limit, territories are directly reformed from the school. In the observation data, it is known that the actual critical limit in increasing phase is in between 4.1 [fish/m^2^] and 5.5 [fish/m^2^], while that limit for decreasing phase is nearly equal to 1.5 [fish/m^2^] (see Tables S1 and S2). From the above, the different phase transition between these increasing and decreasing processes denotes the historical effect which has two transition densities shown to greatly differ^15^. The historical effect is well known in physics, such as ice-water transition and magnetism. However, biological hysteresis is rather rare^16,17^.

In our previous study, we demonstrated the historical effect in ayu fish from the viewpoint of a cost-benefit theory^15^. However, we did not focus on the problems of the population dynamics within the territorial competition as well as the difference between two critical densities of territorial hysteresis. In the present study, to more thoroughly understand the historical effect in the territoriality of ayu, we develop a rate equation to describe the population dynamics within the territorial competition^18^. Our model successfully reproduces territorial hysteresis and indicates that territory holders and floaters can coexist only during the process of population growth. Moreover, we also find that the two critical densities are conspicuously different from each other when the increase of the density of floaters sharply influences (step-function-like) the territories.

## Models and Analyses

### Rate Equation for Territorial Competition

We develop a rate equation to describe the population dynamics of ayu fish within the territorial competition. The territorial competition of ayu fish occurs in the midstream of the river. We set *y* as the proportion of ayu fish to total ayu population and *x* as the proportion of vacant sites to total habitat. Individual fish takes one of two strategies: territory holder (Th) or floater (Fl), and empty sites can be classified into rapids and pools. We define the proportion of territory holder *y*_Th_ and floater *y*_Fl_. For empty sites, we define the proportion of rapids *x*_rapid_ and pools *x*_pool_. They satisfy the conditions: *y* = *y*_Th_ + *y*_Fl_, *x* = *x*_rapid_ + *x*_pool_. Based on many previous studies which describe ecological features of ayu fish^8,11,12,19–21^, we assume that each proportion *y*_Th_, *y*_Fl_, *x*_rapid_ and *x*_pool_ changes over time depending on the following cases:

(i) *y*_Th_:
  increases when floaters find vacant rapids and become territory holders
  decreases when territory holders give up their own territories and become floaters
  decreases when territory holders have died and the places become vacant
(ii) *y*_Fl_:
  decreases when floaters find vacant rapids and become territory holders
  increases when territory holders give up their own territories and become floaters
  decreases when floaters have died and the places become vacant
  increases when newcomers which migrate from downstream become floaters

(iii) *x*_rapid_ increases or decreases by the change of *y*_Th_.
(iv) *x*_pool_ increases or decreases by the change of *y*_Fl_.

**Figure 1:**
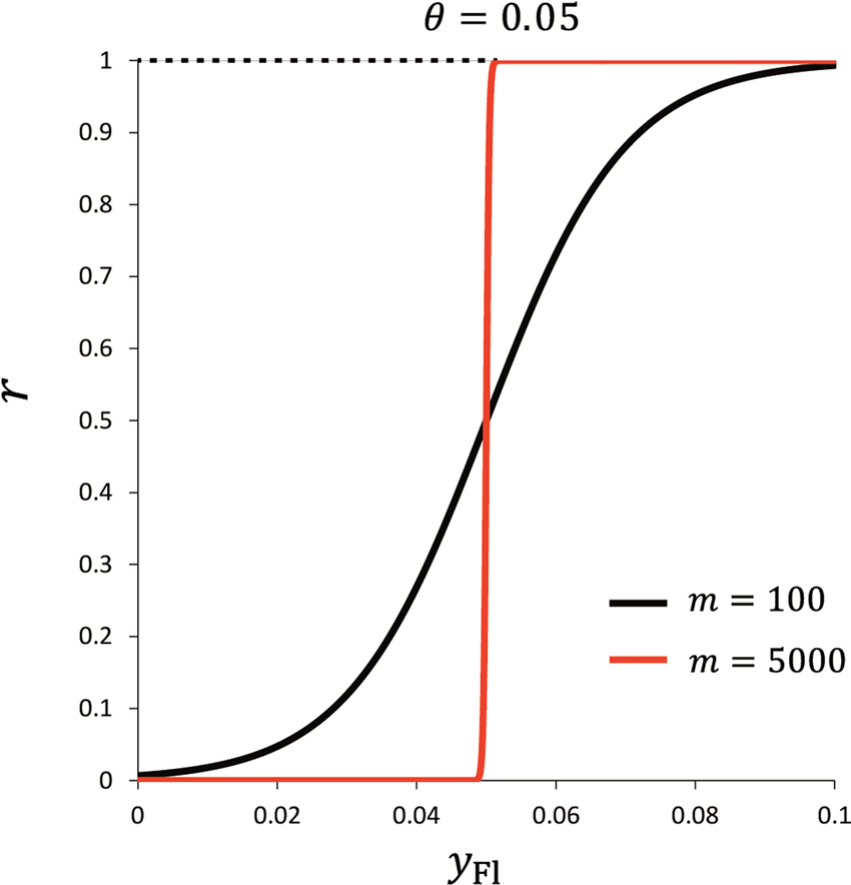
Examples of the function *r*. The function *r* in Eq. (2) indicates the probability that territory holders give up their own territories. This function depends on *y*_Fl_ and two parameters *m* and *θ*, where *m* and *θ* denote the increasing gradient of *r* as a function of *y*_Fl_ and the inflection point, respectively. *r*(*y*_Fl_) represents a logistic curve which has the inflection point *θ* and converges towards 1 as *y*_Fl_ increases (black line). When *m* is sufficiently large, *r*(*y*_Fl_) approximates a step function which has the threshold *θ* (red line).

Corresponding to the cases of (i)–(iv), we obtain the following rate equations if we assume an infinite population, which can be described by the mean field theory.

1 $$\{\begin{array}{rcl}{y}_{{\rm{Th}}}(t+1) & = & {y}_{{\rm{Fl}}}(t)\,{x}_{{\rm{rapid}}}(t)-r[{y}_{{\rm{Fl}}}(t)]{y}_{{\rm{Th}}}(t)-{d}_{{\rm{Th}}}{y}_{{\rm{Th}}}(t)+{y}_{{\rm{Th}}}(t)\quad \quad \quad \,\,\,({\rm{1a}})\\ {y}_{{\rm{Fl}}}(t+1) & = & -{y}_{{\rm{Fl}}}(t){x}_{{\rm{rapid}}}(t)+r[{y}_{{\rm{Fl}}}(t)]{y}_{{\rm{Th}}}(t)-{d}_{{\rm{Fl}}}{y}_{{\rm{Fl}}}(t)+v+{y}_{{\rm{Fl}}}(t)\quad \,\,\,\,\,({\rm{1b}})\\ {x}_{{\rm{rapid}}}(t+1) & = & -{\rm{\Delta }}{y}_{{\rm{Th}}}+{x}_{{\rm{rapid}}}(t)\quad \quad \quad \quad \quad \quad \quad \quad \quad \quad \quad \quad \quad \quad \quad \quad \quad \,\,\,({\rm{1c}})\\ {x}_{{\rm{pool}}}(t+1) & = & -{\rm{\Delta }}{y}_{{\rm{Fl}}}+{x}_{{\rm{pool}}}(t)\quad \quad \quad \quad \quad \quad \quad \quad \quad \quad \quad \quad \quad \quad \quad \quad \quad \quad \,({\rm{1d}})\end{array}$$

where *r* and *v* denote the rate that territory holders give up their own territories and the amount of newcomers which migrate from downstream, respectively. *d*_Th_ and *d*_Fl_ denote the mortality rate of territory holders and floaters. Δ*y*_Th_ and Δ*y*_Fl_ are the temporal variation of *y*_Th_ (=*y*_Th_(*t* + 1) − *y*_Th_(*t*)) and *y*_Fl_ (=*y*_Fl_(*t* + 1) − *y*_Fl_(*t*)), respectively. When the proportion of floaters increases, the defense costs to protect a territory become larger, and territory holders tend to give up their own territories^9,11,12,22^. Thus, we define the function *r* that depends on *y*_Fl_ as follows:

2 $$r({y}_{{\rm{Fl}}})=\frac{1}{1+{e}^{m(\theta -{y}_{{\rm{Fl}}})}}$$

where *m* and *θ* denote the increasing gradient of *r* as a function of *y*_Fl_ and the inflection point, respectively. This function *r*(*y*_Fl_) represents a logistic curve which has the inflection point *θ* and converges towards 1 as *y*_Fl_ increases (Fig. 1). When *m* is sufficiently large, the *r*(*y*_Fl_) approximates a step function which has the threshold *θ*.

We put some limitations on *y*_Th_, *y*_Fl_, *x*_rapid_ and *x*_pool_ based on the actual evidences as follows. In a typical Japanese river, midstream consists of about 55% rapids and about 45% pools^12^. Among these rapids, territory sites that have algae for feeding are estimated to be about 20%. Thus, we assume that rapids which are suitable for territory are about 10% (0.55 × 0.20 = 0.11) of the whole. *y*_Th_ becomes 0.1 when they occupy all rapids (where *x*_rapid_ = 0). In contrast, *y*_Th_ becomes 0 when no one has rapids (where *x*_rapid_ = 0.1). Thus, *y*_Th_ + *x*_rapid_ is always 0.1. Similarly, *y*_Fl_ becomes 0.9 when they occupy all pools (where *x*_pool_ = 0) while *y*_Fl_ becomes 0 when no one has pools (where *x*_pool_ = 0.9). Thus, *y*_Fl_ + *x*_pool_ is always 0.9. Based on the above relationships, *y*_Th_, *y*_Fl_, *x*_rapid_ and *x*_pool_ are:

3 $$\{\begin{array}{c}{y}_{{\rm{Th}}}+{x}_{{\rm{rapid}}}=0.1\\ {y}_{{\rm{Fl}}}+{x}_{{\rm{pool}}}=0.9\\ {y}_{{\rm{Th}}}+{y}_{{\rm{Fl}}}+{x}_{{\rm{rapid}}}+{x}_{{\rm{pool}}}=1\end{array}$$

### Population Dynamics

**Figure 2:**
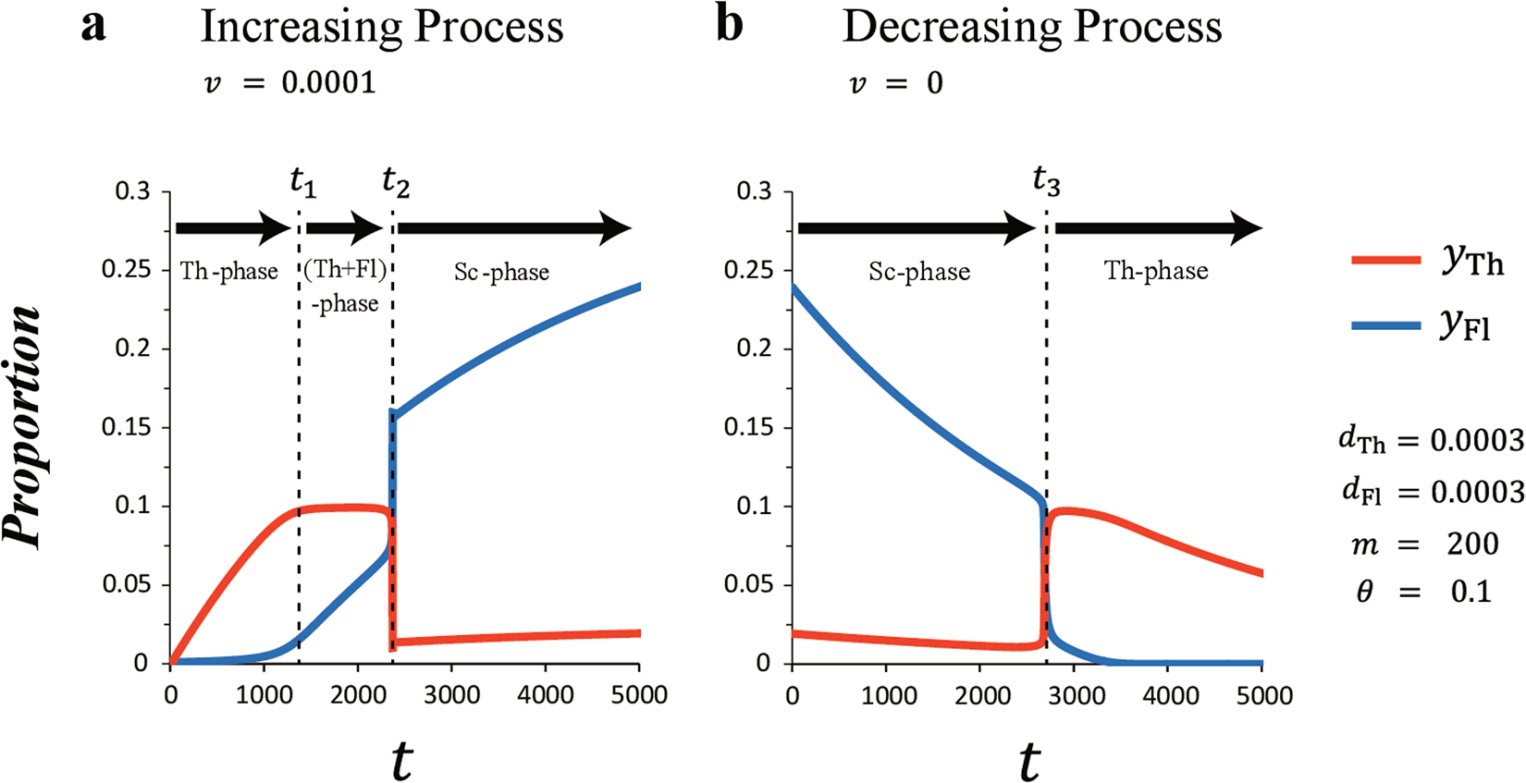
Population dynamics of territory holders and floaters within territorial competition. Model parameters are *d*_Th_ = 0.0003, *d*_Fl_ = 0.0003, *m* = 200 and *θ* = 0.1. The time step *t* is arbitrary. The red (blue) lines denote the proportion of territory holders (floaters). (**a**) Result in increasing stage. In this process, *y*_Th_(0) = *y*_Fl_(0) = 0 and *v* = 0.0001. The final time step *T* is 5000. The phases transit as Th → (Th + Fl) → Sc as follows. When 0 < *t* < *t*_1_, all fish can hold their own territories in rapids at low proportion. When *t*_1_ < *t* < *t*_2_, all territory sites are occupied (*y*_Th_ = 0.1), and surplus fish become floaters. When *t*_2_ < *t*, all fish give up their own territories and form a school. (**b**) Result in decreasing stage. In this process, *y*_Th|dec._(0) = *y*_Th|inc._(*T*), *y*_Fl|dec._(0) = *y*_Fl|inc._(*T*) and *v* = 0. The phases transit as Sc → Th as follows. When 0 < *t* < *t*_3_, all fish are floaters and cannot hold their own territories. However, when *t*_3_ < *t*, territories are directly reformed from the state of a school.

By the use of the above model, we demonstrate the population dynamics of territory holders and floaters within territorial competition between the increasing and decreasing stages of proportion (Fig. 2). In Fig. 2a, the proportion of ayu fish increases due to newcomers migrating from downstream. Thus, the model parameter *v* means that the amount of newcomers is larger than 0. We assume that both initial proportions of territory holders and floaters are 0 (*y*_Th_(0) = *y*_Fl_(0) = 0). This setting means that no fish have migrated to midstream yet, as observed in the state of ayu before spring. We simulate the population dynamics in the increase process where time proceeds from 0 to *T* (Fig. 2a). Here, we set *T* = 5000 as the final time step.

In the increase stage of proportion, the phases transit as Th → (Th + Fl) → School (Sc) gradually (Fig. 2a). When 0 < *t* < *t*_1_, incoming fish can find empty sites among rapids and hold their own territories immediately. In this phase, some territory sites are not occupied (*y*_Th_ < 0.1). When *t*_1_ < *t* < *t*_2_, all territory sites are occupied (*y*_Th_ = 0.1), and surplus fish become floaters. Territory holders and floaters coexist in this phase. When *t*_2_ < *t*, as the proportion of floaters further increases, all fish give up their own territories and form a school (all fish become floaters). In this phase, it is likely that the same territory holders remain in the rapids because the amount does not change. But actually, they are always replaced due to the fact that the amount of territory holders and floaters balances.

In the decreasing process, the proportion of ayu fish decreases by natural death. In this process, we assume that all juvenile fish downstream have already migrated to midstream. Thus, the model parameter *v* is 0. We set the initial proportions of territory holders and floaters equal to the final proportions in the above increasing process. Therefore, the initial proportions are *y*_Th|dec._(0) = *y*_Th|inc._(*T*) and *y*_Fl|dec._(0) = *y*_Fl|inc._(*T*). See *t* = 0 in Fig. 2b. We simulate the population dynamics of the decreasing process until the proportion of ayu becomes sufficiently small. Figure 2b shows part of the simulation (until 5000 time steps) because further steps are redundant.

**Figure 3:**
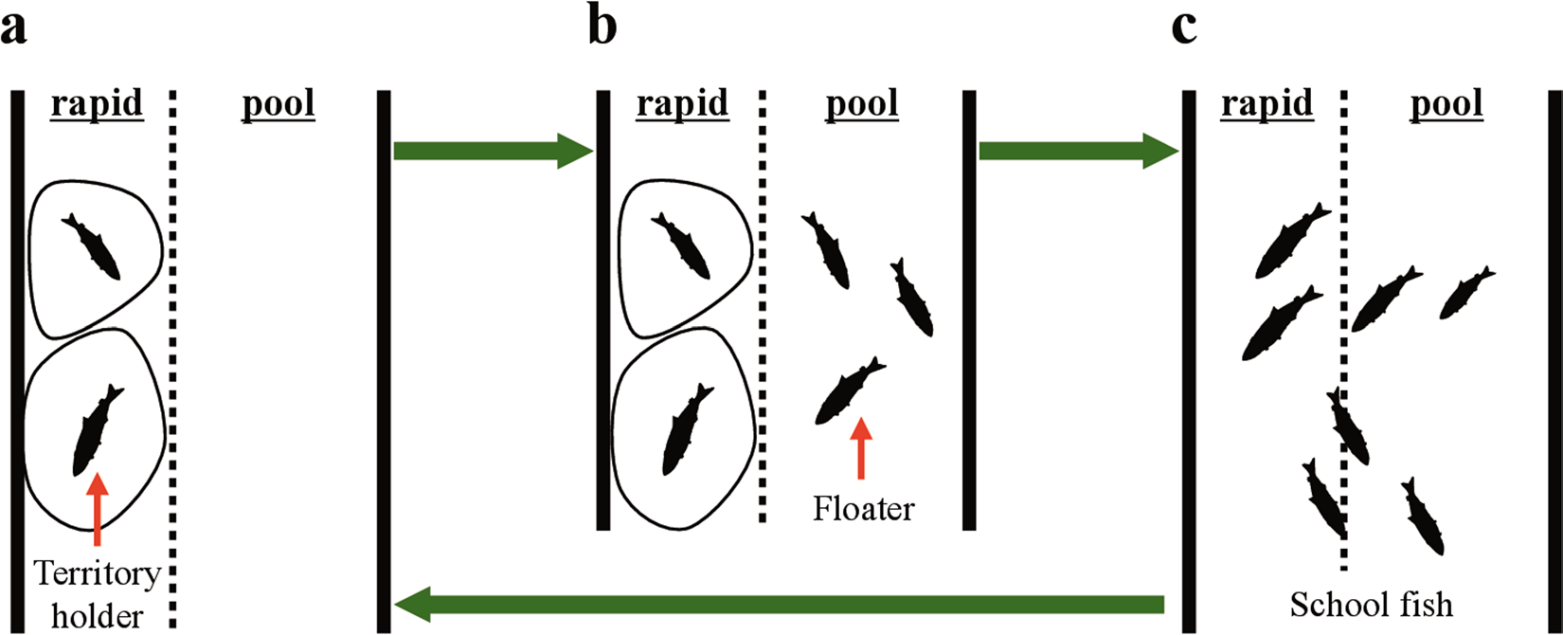
Breakdown and formation processes of territoriality. As the proportion of fish increases, the phases transit as a → b → c. (**a**) In this Th-phase, all fish can hold their own territories in rapids at low proportion, and some territory sites are not occupied. (**b**) In this (Th + Fl)-phase (coexisting phase), when the population size exceeds the territory capacity, newcomers cannot form territories and become floaters in pools. (**c**) In this Sc-phase, as the proportion of fish further increases and exceeds the critical limit, all fish give up their own territories and form a school. In contrast, when the proportion of fish decreases, the phases directly transit as c → a. In other words, when the proportion falls below the critical limit, territories are directly reformed from a school. In contrast to the increasing process, the coexisting phase does not appear in the decreasing process.

In the decrease stage of proportion, the phases transit as Sc → Th (Fig. 2b). When 0 < *t* < *t*_3_, all fish are floaters and cannot hold their own territories. However, when *t*_3_ < *t*, the proportion of fish becomes lower and territories are directly reformed from a school. Amazingly, in contrast to the increasing process (Fig. 2a), the coexisting phase: (Th + Fl)-phase does not appear in the decreasing process. These results mean that territory holders and floaters can coexist together only in the process of the population growth. Figure 3 illustrates the breakdown and formation processes of a territory. Our results indicate that the breakdown and formation processes of territory greatly differ between the increasing and decreasing stages. These results are consistent with studies which describe some ecological features of ayu fish^8–12,19–21^. Moreover, these results of population dynamics are unchanged qualitatively even if the function *r* in equation (2) approximates a step function (1 ≪ *m*) (Fig. S1).

### Historical Effect

**Figure 4:**
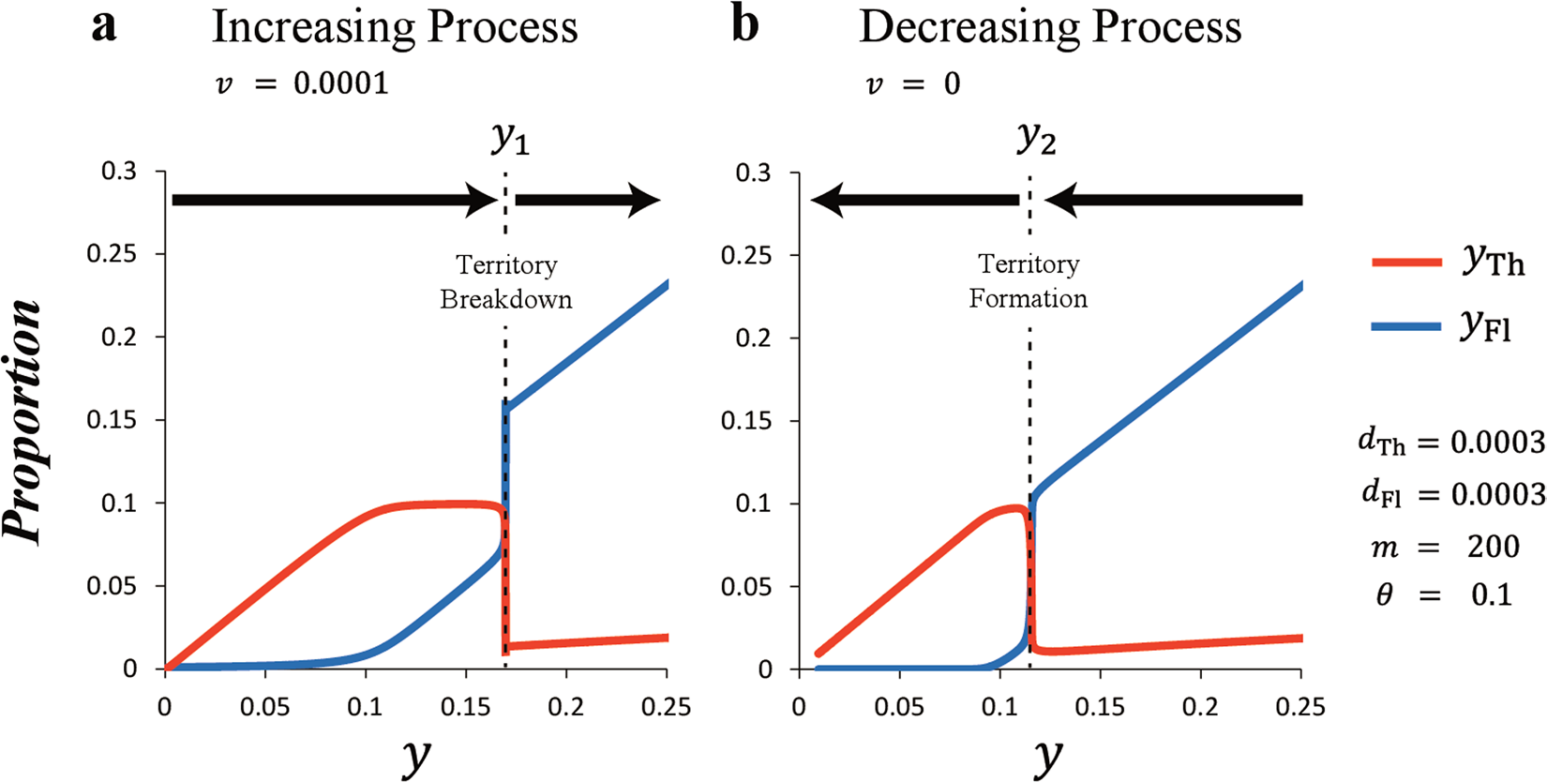
Territorial hysteresis has two significantly different transition proportions. We change the horizontal axis of Fig. 2 from time step *t* to the total proportion of ayu fish *y* (=*y*_Th_ + *y*_Fl_). All model parameters are the same as the conditions used in Fig. 2. The red (blue) lines denote the proportion of territory holders (floaters). (**a**) In the increasing process, when *y* exceeds the critical proportion *y*_1_, all territories break down. (**b**) In the decreasing process, when *N* decreases and falls below the critical proportion *y*_2_, territories are directly reformed from the school. These two transition proportions *y*_1_ and *y*_2_ greatly differ.

We describe the territorial hysteresis which has two significantly different transition proportions (Fig. 4). We change the horizontal axis in Fig. 2 from time step *t* to the total proportion of ayu fish *y* (=*y*_Th_ + *y*_Fl_). Thus, we see the dependence of population dynamics of territory holders and floaters on the total proportion *y* (Fig. 4). All model parameters are the same as the conditions used in the population dynamics. In the increasing process, when *y* exceeds the critical proportion *y*_1_, all territories break down (Fig. 4a). In the decreasing process, when *y* decreases and falls below the critical proportion *y*_2_, territories are directly reformed from the school (Fig. 4b). The hysteresis that is the large difference between *y*_1_ and *y*_2_ occurs for the following reasons. In the increasing process, the proportion of floaters *y*_Fl_ increases after all territory sites are occupied by territory holders. When *y*_Fl_ increases to the inflection point *θ* of the function *r*, it becomes difficult to defend their own territories, and all territories break down. Here, *y*_1_ indicates the sum of proportions of territory holders and floaters. In contrast, in the decreasing process, territory holders do not have their own territories. When *y*_Fl_ decreases to the inflection point *θ* of the function *r*, it becomes easy to reform their own territories, and territories are reformed from the school. Here, *y*_2_ becomes the proportions of only floaters because there are no territory holders in rapids. Due to the above relationships, critical proportions *y*_1_ and *y*_2_ are widely apart. These results of hysteresis are unchanged qualitatively even if the function *r* in equation (2) approximates a step function (1 ≪ *m*) (Fig. S2).

### Sensitivity Analysis

**Figure 5:**
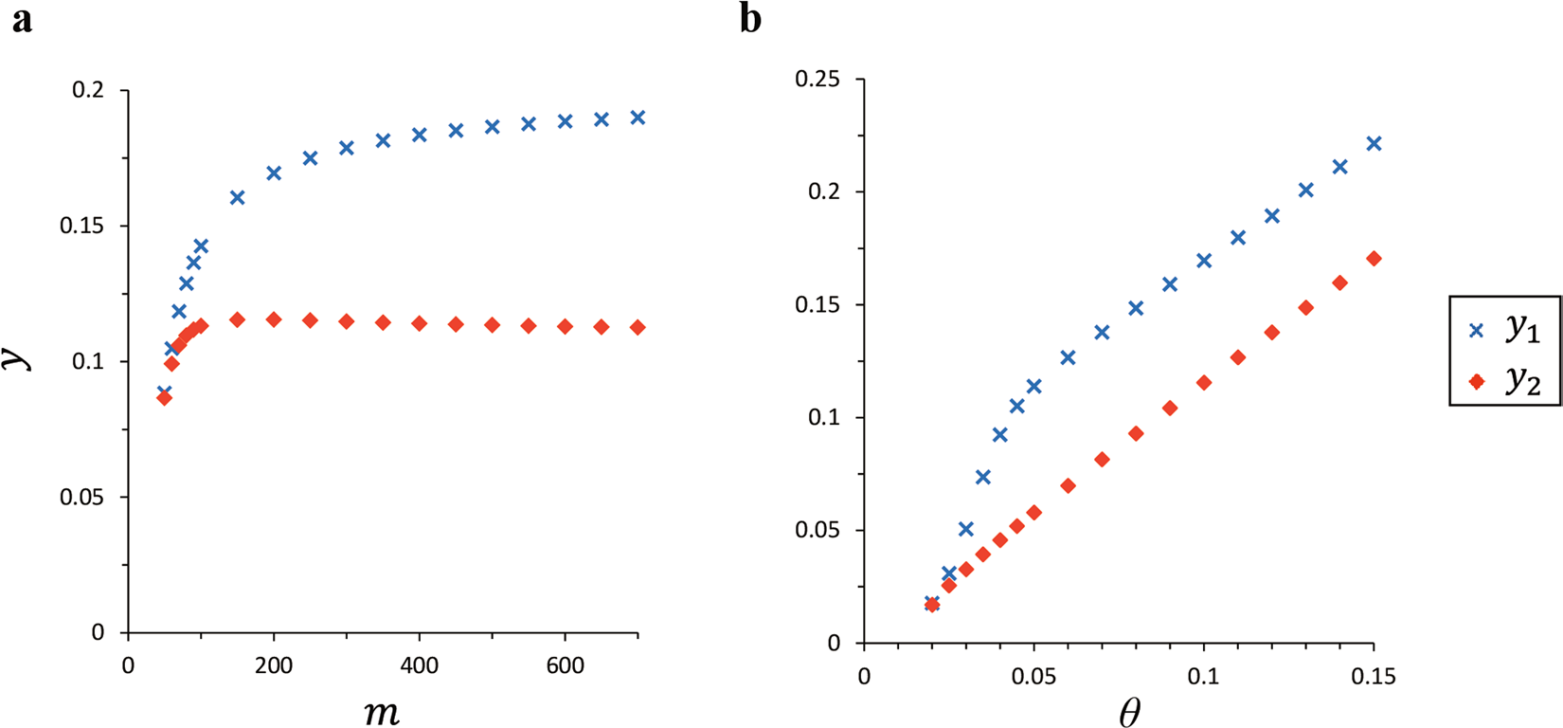
Sensitivity analysis of *y*_1_ and *y*_2_. The blue cross and red diamond denote the transition proportion of territory breakdown *y*_1_ and formation *y*_2_, respectively. (**a**) *y*_1_ and *y*_2_ depending on *m*. All the model parameters excluding *m* are the same as the conditions of the population dynamics in Fig. 2 and hysteresis in Fig. 4. As the model parameter *m* becomes larger, the difference between *y*_1_ and *y*_2_ becomes larger. (**b**) *y*_1_ and *y*_2_ depending on *θ*. All the model parameters excluding *θ* are the same as the conditions of the population dynamics in Fig. 2 and hysteresis in Fig. 4. As the model parameter *θ* becomes larger, the difference between *y*_1_ and *y*_2_ becomes larger.

We explore the sensitivity of critical proportions *y*_1_ and *y*_2_ by changing the parameter *m* while keeping the others are the same as the conditions of the population dynamics in Fig. 2 and hysteresis in Fig. 4 (Fig. 5a). When *m* is sufficiently small, the function *r*(*y*_Fl_) draws a quite gentle curve (Fig. 1). In this case, since territory holders give up their own territories at low proportion of floaters, the phase transition of territory breakdown occurs even if territory sites are not fully occupied by territory holders. Therefore, *y*_1_ and *y*_2_ are not widely apart when *m* is sufficiently small. In contrast, when *m* is large, the function *r*(*y*_Fl_) almost becomes like a step function. In this case, since most territory holders can hold their own territories until the proportion of floater *y*_Fl_ reaches the inflection point *θ*, the phase transition that territory breakdown occurs when all territory sites are occupied. Therefore, *y*_1_ and *y*_2_ are widely apart each other when *m* is large.

Next, we change the parameter *θ* while keeping other model parameters are the same. When *θ* is sufficiently small, the inflection point of function *r*(*y*_Fl_) is small. In this case, since *y*_Fl_ reaches *θ* by the small amount of newcomers *v* and the accumulation of floaters that has not found a territory yet, the phase transition of territory breakdown occurs even if territory sites are not fully occupied by territory holders. Therefore, *y*_1_ and *y*_2_ are not widely apart when *θ* is sufficiently small. In contrast, when *θ* is large, the inflection point of function *r*(*y*_Fl_) becomes large. In this case, since *y*_Fl_ does not reach *θ* until all territory sites are occupied. *y*_1_ and *y*_2_ are widely apart each other when *m* is large.

### Data availability

The authors declare that all data supporting the findings of this study are available within the article and its Supplementary Information files or from the corresponding author upon reasonable request.

## Discussion

In the present study, we develop a rate equation of the population dynamics of ayu fish within territorial competition. Our model indicates that territory holders and floaters can coexist only in the process of population growth (Figs 2 and 3). This coexistence does not appear in the decreasing process. This is the cause of producing territorial hysteresis which has two significantly different transition proportions (Fig. 4). These results are unchanged qualitatively even if the function *r* in Eq. (2) approximates a step function (1 ≪ *m*) (Figs S1 and S2). Moreover, we also find that the two critical proportions conspicuously differ from each other when the increase of the proportion of floaters sharply influences (step-function-like) the territories (Fig. 5).

Few studies have reported such biological hysteresises^16,17^. Caraco (1980) reported an animal hysteresis of avian flocks^16^. These flocks forage for food in two patches. Flock alarm calls and white tail feathers alert the existence of predators so that individual birds can reduce predator scanning as group size increases. As the population size increases, the incoming birds continue to join a single crowded feeding ground. However, as the density of birds greatly increases, food resources per bird are reduced. In that case, choosing a vacant risky site becomes better than joining the crowded patch. In contrast, when the population size decreases so that birds leave the feeding ground, the number of birds in both patches becomes much less than the optimal flock size. In this way, the dynamics of the flock sizes are shifted from the optimal flock size (a single transition point) depending on the situations. Here we demonstrate territoriality of ayu as a significant case of animal hysteresis.

Ayu territory holders are important for biodiversity in a river ecosystem because they specifically feed on algae^23–25^. In recent years, because many rivers are divided by dams, the ayu cannot migrate to midstream, so many fishery cooperative associations actively release ayu into rivers in Japan. We demonstrated that the population dynamics of territory holders and floaters are different between the increasing and decreasing processes due to hysteresis. Thus, those who release ayu should take into account the effect in order to keep the proper number of territory holders. We believe that our model is useful in designing more effective release policies.

## Electronic supplementary material

Supplementary Information

Numerical Simulation

## References

[1] Davies, N. B., Krebs, J. R. & West, S. A. *An Introduction to Behavioral Ecology*, *4th Edition* (Blackwell, Oxford, 2012).

[2] Foster SA (1985). Group foraging by a coral reef fish: a mechanism for gaining access to defended resources. Animal Behavior.

[3] Brown JL, Orians GH (1970). Spacing patterns in mobile animals. Animal Review of Ecology and Systematics.

[4] Ebersole JP (1977). The adaptive significance of interspecific territoriality in the reef fish *Eupomacentrus leucosticus*. Ecology.

[5] Davies, N. B. & Houston, A. I. *Territory economics* (Blackwell Scientific Publications, Oxford, 1984).

[6] Schaller, G. B. *The Serengeti Lion: A Study of Predator-Prey Relations* (Chicago University Press, Chicago, 1972).

[7] Robertson DR, Sweatman HPA, Fletcher EA, Cleland MG (1976). Schooling as a mechanism for circumventing the territoriality of competitors. Ecology.

[8] Kawanabe, H. *Kawa-to-Mizuumi-no-Sakanatati* (Fishes in Rivers and Lakes) (Chuo Koronsha, Tokyo, 1969).

[9] Miyadi, D. *Ayu-no-hanashi* (Stories of Ayu) (Iwanami-shoten, Tokyo, 1960).

[10] Takahashi, K. & Azuma, K. *Kokomade Wakatta Ayu No Hon* (The Up-to-now Knowledge Book of Ayu) (Tsukiji-shoten, Tokyo, 2006).

[11] Iguchi, K. “The territory of Ayu” revisited. *Gekkan kaiyo* **28**, 281–285 (1996).

[12] Kawanabe, H. What is the ‘Nawabari (territory)’ of ayu?: an attempt for the theory of community. *Kagaku* **43**, 74–83 (1973).

[13] Biggs BJF, Hickey CW (1994). Periphyton responses to a hydraulic gradient in a regulated river in New Zealand. Freshwater Biology.

[14] Biggs BJF, Goring DG, Nikora VI (1998). Subsidy and stress responses of stream periphyton to gradients in water velocity as a function of community growth rate. Journal of Phycology.

[15] Tanaka Y, Iguchi K, Yoshimura J, Nakagiri N, Tainaka K (2011). Historical effect in the territoriality of ayu fish. Journal of Theoretical Biology.

[16] Caraco T (1980). Stochastic dynamics of avian foraging flocks. The American Naturalist.

[17] Ronce O, Kirkpatrick M (2001). When sources become sinks: migrational meltdown in heterogeneous habitats. Evolution.

[18] Iwata S, Kobayashi K, Higa S, Yoshimura J, Tainaka K (2011). A simple population theory for mutualism by the use of lattice gas model. Ecological Modelling.

[19] Kawanabe, H. On the significance of the social structure for the mode of density effect in a salmon-like fish, “Ayu”, Plecoglossus altivelis Temminkck et Schlegel. *Memoirs of the College of Science*, *University of Kyoto*, *Series B* **25**, 171–180 (1958).

[20] Kawanabe, H. Social behaviour and production of ayu-fish in the River Ukawa between 1955 and 1969, with reference to the stability of its territoriality. *Japanese Journal of Ecology* **20**, 144–151 (1970).

[21] Katano O, Uchida K, Aonuma Y (2004). Experimental analysis of the territorial establishment of ayu. Plecoglossus altivelis. Ecological Research.

[22] Iguchi K, Hino T (1996). Effect of competitor abundance on feeding territoriality in a grazing fish, the ayu *Plecoglossus altivelis*. Ecological Research.

[23] Katano O, Abe S, Nakamura T (2006). Relationships between ayu *Plecoglossus altivelis* and other organisms in stream communities. Bulletin of Fisheries Research Agency Supplement.

[24] Katano O, Abe S, Matsuzaki K, Iguchi K (2000). Interspecific interactions between ayu, *Plecoglossus altivelis*, and pale chub, *Zacco platypus*, in artificial streams. Fisheries Science.

[25] Abe, S., Iguchi, K., Nagumo, T. & Katano, O. Distribution of ayu (*Plecoglossus altivelis*) and benthic algal flora in the Yaku Island, Kagoshima Prefecture, Japan. *The Japanese Journal of Phycology* **53**, 1–6 (2005).

