# Supplementary material for: Territory holders and non-territory holders in Ayu fish coexist only in the population growth process due to hysteresis
Source: Sci Rep. 2017 Dec 1;7:16777. doi: 10.1038/s41598-017-16859-4 (PMC5711913; doi:10.1038/s41598-017-16859-4)
Supplement: Supplementary file 1 — Supplementary Information [file 41598_2017_16859_MOESM1_ESM.pdf]

Supplementary Information

Territory holders and non-territory holders in Ayu fish coexist only in the population growth process due to hysteresis

Yuki Katsumata, Takashi Uehara, Hiromu Ito, Jin Yoshimura, Kei-ichi Tainaka and Genki Ichinose

Supporting Information

1. Observation data of territorial hysteresis of ayu fish

The observation data of fish state in the increasing process are listed in Table S1. In Table S1, territoriality is maintained when the fish density n [fish/m^2] is smaller than 4.1, and schooling is seen when n becomes larger than 5.5. These data suggest that territory breakdowns occur for $4.1<n<5.5$.

Table S1. The evidence of territoriality in ayu fish in the increasing process.

Year	River or pond	Natural or experiment	Density [fish/m^2]	State of fish	Reference
1955	Ukawa	Natural	5.5	School	[1]
1956	Ukawa	Natural	0.9	Territory	[1]
1957	Ukawa	Natural	0.3	Territory	[1]
1958	Inukai	Experiment	4.1	Territory	[1]
1987	Pond	Experiment	195.2	School	[2]
1987	Pond	Experiment	97.6	School	[2]
1989	Pond	Experiment	120.4	School	[2]
1989	Pond	Experiment	25.0	School	[2]
1991	Experimental stream		1.0	Territory	[3]
1991	Experimental stream		0.6	Territory	[3]
1999	Pond	Experiment	1250.0	School	[4]
1999	Pond	Experiment	400.0	School	[4]
1999	Pond	Experiment	100.0	School	[4]

Next, the observation data of fish state in the decreasing process are listed in Table S2. In Table S2, when $n>25.0$, all fish form a school. In contrast, when $n<1.5$, all fish can hold their own territories. When $n=5.0$, the territories are rather unstable and small compared to the increasing process. The phase transition from school to territory clearly occurs when the values of n is

close to 1.5. Thus, it is considered that the critical density of territory breakdown is about 5 [fish/m^2] and territory formation is about 2 [fish/m^2] from the results of experimental stream[5]. As stated above, these critical densities are widely apart each other.

Table S2. The state of fish in the decreasing process.

Year	River or pond	Natural or experiment	Density [fish/m^2]	State of fish	Reference
1958	Inukai	Experiment	1.5	Territory	[1]
1958	Inukai	Experiment	0.5	Territory	[1]
1987	Pond	Experiment	195.2	School	[2]
1987	Pond	Experiment	97.6	School	[2]
1989	Pond	Experiment	120.4	School	[2]
1989	Pond	Experiment	25.0	School	[2]
1991	Experimental stream		5.0	School(Th*)	[3]
1991	Experimental stream		1.5	Territory	[3]
1991	Experimental stream		1.0	Territory	[3]
1991	Experimental stream		0.6	Territory	[3]
1991	Experimental stream		0.3	Territory	[3]

Th*: Attempted territory holders appear: their territories are small and unstable.

2. Change of model parameter *m* to sufficiently large value (1<<*m*)

We show the case that *m* is sufficiently large in the population dynamics (Fig. S1).

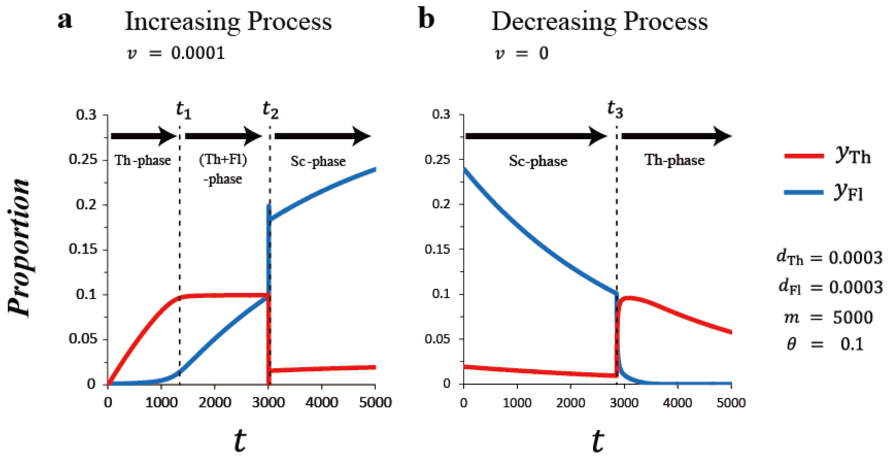

Figure S1. Population dynamics when *m* is sufficiently large. Model parameters are d_{Th} =0.0003, d_{Fl} =0.0003, *m*=5000 and θ=0.1. The time step *t* is arbitrary. The red (blue) lines

denote the proportion of territory holders (floaters). (**a**) Result in increasing stage. In this process, $y_{\mathrm{Th}}(0)=y_{\mathrm{Fl}}(0)=0$ and v=0.0001. The final time step T is 5000. The phases transit as Th→(Th+Fl)→Sc as follows. When $0<t<t_1$, all fish can hold their own territories in rapids at low proportion. When $t_1<t<t_2$, all territory sites are occupied (y_{Th}=0.1), and surplus fish become floaters. When $t_2<t$, all fish give up their own territories and form a school. (**b**) Result in decreasing stage. In this process, $y_{\mathrm{Th|dec.}}(0)= y_{\mathrm{Th|inc.}}(T)$, $y_{\mathrm{Fl|dec.}}(0) = y_{\mathrm{Fl|inc.}}(T)$ and v=0. The phases transit as Sc→Th as follows. When $0<t<t_3$, all fish are floaters and cannot hold their own territories. However, when $t_3<t$, territories are directly reformed from the state of a school. These results are qualitatively equal to Fig. 2.

We show the case that m is sufficiently large in the historical effect (Fig. S2).

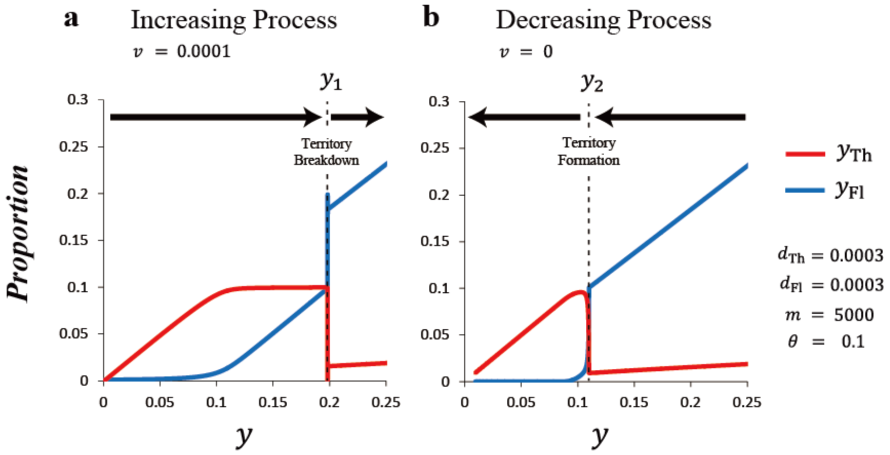

Figure S2. Territorial hysteresis when *m* is sufficiently large. We change the horizontal axis of Fig. S1 from time step t to the total density of ayu fish y ($=y_{\mathrm{Th}}+y_{\mathrm{Fl}}$). All model parameters are the same as the conditions used in Fig. S1. The red (blue) lines denote the proportion of territory holders (floaters). (**a**) In the increasing process, when y exceeds the critical proportion y_1, all territories break down. (**b**) In the decreasing process, when y decreases and falls below the critical density y_2, territories are directly reformed from the school. These two transition densities y_1 and y_2 greatly differ. These results are qualitatively equal to Fig. 4.

References:

1. Kawanabe, H. *Kawa-to-Mizuumi-no-Sakanatati* (Fishes in Rivers and Lakes) (Chuo Koronsha, Tokyo, 1969).
2. Iguchi, K. & Yamaguchi, M. Adaptive significance of inter- and intra- populational egg size variation in ayu *Plecoglossus altivelis* (Osmeridae). *Copeia*, 184-190 (1994).
3. Iguchi, K. “The territory of Ayu” revisited. *Gekkan kaiyo* **28**, 281-285 (1996) (in Japanese).

4. Iguchi, K., Ogawa, K., Nagae, M. & Ito, F. The influence of rearing density on stress response and disease susceptibility of ayu (*Plecoglossus altivelis*). *Aquaculture* **220**, 515-523 (2003).
5. Iguchi, K. (private communication).
